# Risk of Bias Mitigation for Vulnerable and Diverse Groups in Community-Based Primary Health Care Artificial Intelligence Models: Protocol for a Rapid Review

**DOI:** 10.2196/46684

**Published:** 2023-06-26

**Authors:** Maxime Sasseville, Steven Ouellet, Caroline Rhéaume, Vincent Couture, Philippe Després, Jean-Sébastien Paquette, Karine Gentelet, David Darmon, Frédéric Bergeron, Marie-Pierre Gagnon

**Affiliations:** 1 Faculté des sciences infirmières Université Laval Quebec, QC Canada; 2 VITAM Research Center on Sustainable Health Quebec, QC Canada; 3 Faculté de médecine Université Laval Quebec, QC Canada; 4 Département de physique, de génie physique et d'optique Université Laval Quebec, QC Canada; 5 Département des sciences sociales Université du Québec en Outaouais Gatineau, QC Canada; 6 Direction du service et du centre de santé universitaire Université Côte d'Azur Nice France; 7 Bibliothèque – Direction des services-conseils Université Laval Quebec, QC Canada

**Keywords:** health equity, health disparity, algorithms, artificial intelligence, primary care, rapid review, systematic review

## Abstract

**Background:**

The current literature identifies several potential benefits of artificial intelligence models for populations’ health and health care systems' efficiency. However, there is a lack of understanding on how the risk of bias is considered in the development of primary health care and community health service artificial intelligence algorithms and to what extent they perpetuate or introduce potential biases toward groups that could be considered vulnerable in terms of their characteristics. To the best of our knowledge, no reviews are currently available to identify relevant methods to assess the risk of bias in these algorithms. The primary research question of this review is which strategies can assess the risk of bias in primary health care algorithms toward vulnerable or diverse groups?

**Objective:**

This review aims to identify relevant methods to assess the risk of bias toward vulnerable or diverse groups in the development or deployment of algorithms in community-based primary health care and mitigation interventions deployed to promote and increase equity, diversity, and inclusion. This review looks at what attempts to mitigate bias have been documented and which vulnerable or diverse groups have been considered.

**Methods:**

A rapid systematic review of the scientific literature will be conducted. In November 2022, an information specialist developed a specific search strategy based on the main concepts of our primary review question in 4 relevant databases in the last 5 years. We completed the search strategy in December 2022, and 1022 sources were identified. Since February 2023, two reviewers independently screened the titles and abstracts on the Covidence systematic review software. Conflicts are solved through consensus and discussion with a senior researcher. We include all studies on methods developed or tested to assess the risk of bias in algorithms that are relevant in community-based primary health care.

**Results:**

In early May 2023, almost 47% (479/1022) of the titles and abstracts have been screened. We completed this first stage in May 2023. In June and July 2023, two reviewers will independently apply the same criteria to full texts, and all exclusion motives will be recorded. Data from selected studies will be extracted using a validated grid in August and analyzed in September 2023. Results will be presented using structured qualitative narrative summaries and submitted for publication by the end of 2023.

**Conclusions:**

The approach to identifying methods and target populations of this review is primarily qualitative. However, we will consider a meta-analysis if quantitative data and results are sufficient. This review will develop structured qualitative summaries of strategies to mitigate bias toward vulnerable populations and diverse groups in artificial intelligence models. This could be useful to researchers and other stakeholders to identify potential sources of bias in algorithms and try to reduce or eliminate them.

**Trial Registration:**

OSF Registries qbph8; https://osf.io/qbph8

**International Registered Report Identifier (IRRID):**

DERR1-10.2196/46684

## Introduction

### Background

There is growing interest in artificial intelligence (AI) in medical research and clinical practice. Judicious use of algorithms could potentially improve the quality of care and accessibility to health services [1,2]. AI in health care is mostly used for training supervised models on previously collected data [1,3]. Some health specialties benefit from these tools that allow the application of AI models safely with a controlled risk of bias. For instance, the use of AI in medical imaging allows an improvement in the detection of some anomalies [4,5], and initiatives in radiology seem to improve efficiency and health outcomes [6]. Similarly, AI models could potentially have a positive impact on populations in vulnerable situations who access the health care system through primary care [1,7]. Unfortunately, AI remains poorly integrated into primary and community health services [8].

A potential use and implementation of AI models in the primary health care context could be in citizen portals by promoting health-oriented behaviors and preventing the onset of chronic diseases [8]. As elsewhere in Canada and worldwide, such portals are currently implemented in some electronic medical records in the province of Québec and serve as an interface between clinicians and patients [9]. These portals can provide access to laboratory results, reports of health questionnaires, or communication with health care professionals.

Citizen portals with integrated AI could lead to virtual assistance throughout the health care system or in situations where vulnerable populations do not have direct and rapid access to physicians’ advice (eg, remote, isolated, vulnerable, and stigmatized populations) [1,2]. However, since these AI models are deployed in communities and vulnerable populations are concerned, there are risks of biases affecting patient care quality and safety, and their sources must be explored and identified prior to implementation [10]. We need to take a specific interest in medical and ethical parameters to ensure user quality and safety, as these models may be biased especially by ethnicity, sex, or gender [11-14]. Moreover, inequitable access to resources and recommendations could potentially put some users at risk [10].

### Community-Based Primary Health Care

As defined by the Canadian Institutes of Health Research, community-based primary health care (CBPHC) “covers the broad range of primary prevention (including public health) and primary care services within the community, including health promotion and disease prevention; the diagnosis, treatment, and management of chronic and episodic illness; rehabilitation support; and end of life care” [15]. The current literature identifies several potential benefits of AI to develop and integrate clinical tools for population health, care team well-being, and efficiency of the CBPHC system [1,16-18]. Integration of AI technologies into CBPHC could help in many ways especially “to help people to get the care (including prevention) they need” [15]. However, there is a lack of understanding on how the risk of bias is considered in the development of these AI models and to what extent they perpetuate or introduce potential biases toward groups that could be considered vulnerable in terms of their characteristics (eg, sex, gender identity, sexual orientation, race, ethnicity, socioeconomic status) [16,19]. In response to this concern, the US Food and Drug Administration recently “calls for efforts to develop a methodology to develop devices to be well suited for a racially and ethnically diverse patient population” [1,16].

In a recent scoping review with the objective to “assess the nature and extent of the body of research on AI to primary care,” the authors concluded that AI “is at an early stage of maturity (...) and more evaluation studies are needed” [7]. To the best of our knowledge, no reviews are available on methods to assess risk of bias and health inequity in primary care algorithms. We recently found a scoping review protocol aiming “to summarize the extent to which AI systems in primary care examine the inherent bias toward or against vulnerable populations and appraise how these systems have mitigated the impact of such biases during their development” [20]. Unless we are mistaken, the results of this protocol have not yet been published, and we still lack an overview of bias mitigation interventions and the characterization of groups and subgroups to which they are applied in CBPHC.

The aim of this rapid qualitative systematic review is to identify relevant methods (eg, frameworks, tools, checklists) for a risk of bias assessment for vulnerable or diverse groups in the development or deployment of algorithms in CBPHC and identify mitigation strategies deployed to promote and increase equity, diversity, and inclusion in CBPHC algorithms.

### Review Questions

The research questions are:

- Which strategies have been suggested for consideration when assessing the risk of bias in CBPHC algorithms toward vulnerable or diverse groups?
- What methods assessed the risk of bias for vulnerable and diverse groups in CBPHC algorithm applications?
- Which mitigation interventions are deployed and (if applicable) what are the quantitative results?

## Methods

### Overview

This protocol follows the Cochrane Collaboration guidance for rapid reviews [21] and the PRISMA-P (Preferred Reporting Items for Systematic Review and Meta-Analysis Protocols) guidelines [22,23]. PRISMA-P guidelines recommend the use of PICO (Participant, Intervention, Comparator, and Outcome) to frame the review question and develop a search strategy [22,23]. However, other options are available depending on the review objective. For a scoping review, the Joanna Briggs Institute recommends the PCC (Population or Participant, Concept, and Context) search framework [24]. Although we are not in a scoping review process, the PCC search framework [25] seems more appropriate and consistent with our open-ended qualitative research question (Table 1).

**Table 1:** PCC (Population or Participant, Concept, and Context) framework used for the search strategy^a^.

PCC elements	Definition (per JBI Reviewer’s Manual Ch 11)	PCC elements applied in this review
Population	“Important characteristics of participants, including age and other qualifying criteria” (11.2.4)	Any vulnerable populations or diverse groups
Concept	“The core concept examined by the scoping review should be clearly articulated to guide the scope and breadth of the inquiry. This may include details that pertain to elements that would be detailed in a standard systematic review, such as the ‘interventions’ and/or ‘phenomena of interest’ and/or ‘outcomes’“ (11.2.4)	Strategies or methods to assess risk of bias in algorithms (artificial intelligence)
Context	“May include...cultural factors such as geographic location and/or specific racial or gender-based interests. In some cases, context may also encompass details about the specific setting.”	Community-based primary health care

^a^PCC framework [25]. Topic: risk of bias assessment methods developed/used in community-based primary health care algorithms. Primary review question: Which strategies can assess the risk of bias in community-based primary health care algorithms toward vulnerable or diversity groups?

### Search Strategy

In November 2022, an information specialist developed a search strategy based on the main concepts of our primary review question and adapted it to the most relevant databases (PubMed, CINAHL, Web of Science, and PsychINFO). We used five relevant articles to test the sensitivity, and the search covered the last 5 years (2017-2022) aligned with the concept’s emergence time frame. The database search strategies can be found in Multimedia Appendix 1.

### Data Collection

We include all studies on methods developed or tested to assess risk of bias in algorithms that are relevant in CBPHC. In December 2022, all citations were exported to the web-based collaborative tool Covidence (Veritas Health Innovation) [26] where 581 duplicates were removed by the automated function. Since February 2023, two reviewers have independently screened the titles and abstracts of the identified records (N=1022) and assessed the inclusion and exclusion criteria (Table 2). Conflicts on study selection are resolved by consensus, and the opinion of a senior researcher is sought if required. At the full-text assessment step, two reviewers (and the information specialist if required) will search and obtain all the full texts of the selected references, and they will import the PDF files into Covidence.

**Table 2:** Inclusion and exclusion criteria.

PCC^a^ elements [25]	Inclusion criteria	Exclusion criteria
Population	Any vulnerable populations or diverse groups targeted by CBPHC^b^ interventions	Any populations targeted by hospital or specialized care interventions
Concept	All the methods, tools, recommendations, or any intervention used to assess the risk of bias in CBPHC algorithms All mitigation strategies deployed to promote and increase equity, diversity, and inclusion in CBPHC algorithms	Methods or interventions not CBPHC related CBPHC interventions that do not include any algorithm/AI^c^ system
Context	Include all the CBPHC algorithms (AI) applications that can perpetuate/introduce potential biases toward groups that could be considered as vulnerable in terms of their characteristics	Algorithms used by primary health care providers for support in administrative tasks and for operational aspects, rather than for clinical decisions

^a^PCC: Population or Participant, Concept, and Context.

^b^CBPHC: community-based primary health care.

^c^AI: artificial intelligence.

### Data Extraction

Team members will complete the extraction on a structured prespecified grid in Covidence, and the data will be reviewed and pretested by the research team. We will extract descriptive data (title, year of publication, authors, country), study type (published, study design), and population data (populations targeted by the intervention, number of participants). Characteristics of populations will be mainly extracted to our prespecified grid according to the PROGRESS (place of residence, race/ethnicity/culture/language, occupation, gender/sex, religion, education, socioeconomic status, and social capital) framework [27]. This framework addresses “health inequalities and unfair differences in disease burden,” and these characteristics “that stratify health opportunities and outcomes” are “place of residence, race/ethnicity/culture/language, occupation, gender/sex, religion, education, socioeconomic status, and social capital” [27]. We will add to that list the “Plus” in the acronym PROGRESS-Plus, referring to the “1) personal characteristics associated with discrimination (e.g. age, disability) 2) features of relationships (e.g. smoking parents, excluded from school) 3) time-dependent relationships (e.g. leaving the hospital, respite care, other instances where a person may be temporarily at a disadvantage)” [28].

We will also extract concept data: identification and characteristics of the assessment method used/developed; identification and characteristics of the algorithm, including purpose (eg, diagnosis- or prognosis-related, used for disease detection or surveillance), type (eg, machine learning, natural language processing, expert systems), and any other available detail (eg, input information/parameters, outcomes); and context data, including details about the specific settings (ie, identification of potential biases for vulnerable or diverse groups) and the results of the intervention (eg, bias mitigation data).

### Quality Assessment

We will appraise the quality of empirical studies included based on experiment, observation, or simulation (randomized controlled trial, quasi–randomized controlled trial, prospective cohort study, pre-post study, observational study, mixed methods study, qualitative study) by applying the Mixed Methods Appraisal Tool (MMAT) [29].

### Data Synthesis

We will synthesize data using structured narrative summaries around our main concept/context and provide a list of available methods. The development process and main features of the methods (eg, potential sources of bias/items covered, signaling questions, differences and similarities, strengths and weaknesses) will be described. Furthermore, we will present mitigation strategies deployed with these methods (eg, algorithm parameter adjustments, mitigation or elimination of bias).

### Ethics Approval

We obtained approval by the Ethics board of the “Comité d’éthique de la recherche sectoriel en Santé des Populations et Première Ligne du CIUSSS de la Capitale-Nationale” for the Protecting and Engaging Vulnerable Populations in the Development of Predictive Models in Primary Health Care for Inclusive, Diverse and Equitable AI (PREMIA) project (#2023-2726).

## Results

We completed the search strategy in December 2022, when 1022 sources were identified and 581 duplicates were removed by the automated function of Covidence. In early May 2023, a total of 479 out of 1022 (46.9%) titles and abstracts have been screened. This first screening step was completed in May 2023. In June and July 2023, two reviewers will independently apply the same inclusion and exclusion criteria to full texts. Data from the selected studies will be extracted using a validated grid in August 2023. The quality of studies will be appraised along with the data extraction by applying the MMAT [29] in August 2023. Data will be analyzed in September 2023, and the results will be presented using structured qualitative narrative summaries. We plan to complete this review and submit a final manuscript by the end of 2023.

## Discussion

### Principal Findings

We anticipate structured narrative summaries of identified bias mitigation methods would be useful to researchers, data scientists, ethicists, and other CBPHC providers. We also anticipate that identification and documentation of these methods would be particularly relevant for multidisciplinary teams: “AI initiatives should have two main goals: they should be designed and utilized in a manner that does not create or maintain health disparities currently experienced by vulnerable groups, and they should address and remove existing health disparities” [1]. We hypothesize that giving methods and tools to multidisciplinary teams, with the participation of vulnerable populations in the entire process, would be useful to achieve inclusive AI when applied to health. We also expect that few studies will present quantitative bias mitigation data and results. Nevertheless, we will present these data in qualitative structured summaries.

### Comparison to Prior Work

Other reviews generally focused on a few specific population characteristics. In a recent study on hospital readmission rates, the authors focused on racial and socioeconomic status biases in algorithms due to an increased risk in these groups [19]. In another example, the authors explored the source of age-related bias in AI systems [30]. There are many other “subgroups or unprotected groups” that can be considered to address “health inequalities and unfair differences in disease burden” [31]. We expect that our focus on primary health care will offer a different insight and that the PROGRESS-Plus framework will be helpful to identify potential interventions to mitigate health inequalities [27,28]. We anticipate listing and describing which vulnerable or diverse groups are considered (and which could be considered).

### Strengths and Limitations

The main strength of our review is that we are applying an equity lens [27,28] to describe mitigation strategies in primary health care AI models. However, this review has three main limitations. First, its rapid nature implies that we limit our search to the last 5 years and to 4 databases only. Some relevant studies might not be included. However, given the recent interest in AI in primary care, this is unlikely. Second, we will consider all types of health conditions and various types of populations, which could bring important heterogeneity in the results and limit the possibility of synthesizing this information. Finally, applying the findings of this review to existing AI-based algorithms could present challenges because of the different contexts and populations in which they were developed.

### Future Directions and Dissemination Plan

This rapid review is the first step of the larger PREMIA project. This work will be presented to key stakeholders, and we will conduct qualitative data collection to identify and evaluate biases in one AI model. Giving a voice to diverse groups [32,33] at the development or deployment stages of CBPHC AI-based algorithms could have a substantial impact on this review. Recommendations will be implemented into tools able to guide developers, researchers, and decision makers on strategies to reduce the risk of bias in AI primary health care programs and services, with the objective to protect vulnerable groups and not further exacerbate inequalities.

### Conclusion

Identifying methods developed and deployed to mitigate biases toward vulnerable populations is needed to develop equitable primary health care. AI models can have many benefits for patients, but factors that stratify health opportunities and outcomes need to be addressed to mitigate inequalities toward members of vulnerable or diverse groups. To our knowledge, no reviews are currently available to identify risk of bias mitigation strategies in CBPHC AI algorithms. This rapid review will contribute to providing comprehensive structured narrative summaries of risk of bias assessment methods. This knowledge could be useful to CBPHC researchers and other stakeholders to identify potential sources of bias in AI algorithm development and eventually try to reduce or eliminate them.

## Appendix

Multimedia Appendix 1 The search strategies for each database.

Multimedia Appendix 2 Peer-review reports from the l’Observatoire international sur les impacts sociétaux de l’IA et du numérique (OBVIA) / International Observatory on the Societal Impacts of AI and Digital Technology (Quebec, Canada).

## Abbreviations

AI: artificial intelligence
CBPHC: community-based primary health care
MMAT: Mixed Methods Appraisal Tool
PCC: Population or Participant, Concept, and Context
PICO: Participant, Intervention, Comparator, and Outcome
PREMIA: Protecting and Engaging Vulnerable Populations in the Development of Predictive Models in Primary Health Care for Inclusive, Diverse and Equitable AI
PRISMA-P: Preferred Reporting Items for Systematic Review and Meta-Analysis Protocols
PROGRESS: place of residence, race/ethnicity/culture/language, occupation, gender/sex, religion, education, socioeconomic status, and social capital

## Data Availability

The database search strategy data is available in Multimedia Appendix 1. All data generated or analyzed during this study (including the PRISMA [Preferred Reporting Items for Systematic Reviews and Meta-Analyses] flow diagram) will be included in the results manuscript submission as multimedia appendix files. Databases created in the data extraction process can be provided upon reasonable request.
