# Supplementary material for: Risk of Bias Mitigation for Vulnerable and Diverse Groups in Community-Based Primary Health Care Artificial Intelligence Models: Protocol for a Rapid Review
Source: JMIR Res Protoc. 2023 Jun 26;12:e46684. doi: 10.2196/46684 (PMC10337340; doi:10.2196/46684)
Supplement: Multimedia Appendix 1 [file resprot_v12i1e46684_app1.docx]

**Supplementary materials – Databases search strategy**

**Medline (OVID)**

**Date of the search:** 15-11-2022

**Database limit:** The results of the research strategy were limited to the last 5 years (2017-2022)

| **#** | **Search strategy** | **Results** |
| --- | --- | --- |
| 1 | Artificial Intelligence/ OR exp "Machine Learning"/ OR exp Neural Networks, Computer/ OR Expert Systems/ OR Natural Language Processing/ OR Algorithms/ | 375775 |
| 2 | Expert System?.ti,ab,kw,kf OR ((artificial OR computational) adj2 intelligence).ti,ab,kw,kf OR ((Machine? OR Supervised OR Unsupervised OR Reinforcement OR Deep) adj2 Learning).ti,ab,kw,kf OR "Neural Networks".ti,ab,kw,kf OR (AI adj (technolog* OR system? OR algorith* OR application?)).ti,ab,kw,kf OR (predicti* adj (model? OR algorithm?)).ti,ab,kw,kf OR "natural language processing".ti,ab,kw,kf | 213938 |
| 3 | 1 OR 2 | 504772 |
| 4 | Bias/ | 25839 |
| 5 | Bias.ti,ab,kw,kf OR biases.ti,ab,kw,kf | 239393 |
| 6 | 4 OR 5 | 250499 |
| 7 | Community Health Services/ OR Primary Health Care/ OR Delivery of Health Care/ OR Healthcare Disparities/ OR Health Equity/ OR Decision Support Systems, Clinical/ OR Decision Support Techniques/ | 275373 |
| 8 | "Decision Support".ti,ab,kw,kf OR ((Primary OR Community OR Health) adj2 Care).ti,ab,kw,kf OR ((Health OR delivery) adj3 Care).ti,ab,kw,kf OR Healthcare.ti,ab,kw,kf OR "Health Service?".ti,ab,kw,kf OR (health* adj2 (equit* OR disparit*)).ti,ab,kw,kf | 980094 |
| 9 | 7 OR 8 | 1093443 |
| 10 | 3 AND 6 AND 9 | 825 |
| 11 | limit 10 to yr="2017-2022" | 598 |

**Web of Science**

**Date of the search:** 15-11-2022

**Database limit:** The results of the search strategy were limited to the following 2 index: Science Citation Index Expanded (SCI-EXPANDED) & Emerging Sources Citation Index (ESCI); The results of the research strategy were limited to the last 5 years (2017-2022)

| **#** | **Search strategy** | **Results** |
| --- | --- | --- |
| 1 | TS=("Expert System$") OR TS=((artificial OR computational) NEAR/2 intelligence) OR TS=((Machine$ OR Supervised OR Unsupervised OR Reinforcement OR Deep) NEAR/2 Learning) OR TS=("Neural Networks") OR TS=(AI NEAR/1 (technolog* OR system$ OR algorith* OR application$)) OR TS=(predicti* NEAR/1 (model$ OR algorithm?)) OR TS="natural language processing" | 730,635 |
| 2 | TS=(Bias) OR TS=(biases) | 521,814 |
| 3 | TS=("Decision Support") OR TS=((Primary OR Community OR Health) NEAR/2 Care) OR TS=((Health OR delivery) NEAR/3 Care) OR TS=(Healthcare) OR TS=("Health Service$") OR TS=(health* NEAR/2 (equit* OR disparit*)) | 920,602 |
| 4 | #1 AND #2 AND #3 | 817 |
| 5 | #4 AND PY=(2017-2022) | 695 |

**CINAHL (EBSCO)**

**Date of the search:** 15-11-2022

**Database limit:** The results of the research strategy were limited to the last 5 years (2017-2022)

| **#** | **Search strategy** | **Results** |
| --- | --- | --- |
| 1 | MH "Artificial Intelligence" OR MH "Natural Language Processing" OR MH "Expert Systems" OR MH "Neural Networks (Computer)" OR MH "Machine Learning" OR MH "Deep Learning"OR MH Algorithms OR MH "Prediction Models" | 58,238 |
| 2 | TI "Expert System#" OR AB "Expert System#" OR TI ((artificial OR computational) N2 intelligence) OR AB ((artificial OR computational) N2 intelligence) OR TI ((Machine# OR Supervised OR Unsupervised OR Reinforcement OR Deep) N2 Learning) OR AB ((Machine# OR Supervised OR Unsupervised OR Reinforcement OR Deep) N2 Learning) OR TI "Neural Networks" OR AB "Neural Networks" OR TI (AI N1 (technolog* OR system# OR algorith* OR application#)) OR AB (AI N1 (technolog* OR system# OR algorith* OR application#)) OR TI (predicti* N1 (model# OR algorithm#)) OR AB (predicti* N1 (model# OR algorithm#)) OR TI "natural language processing" OR AB "natural language processing" | 40,870 |
| 3 | S1 OR S2 | 85,816 |
| 4 | MH "Implicit Bias" OR MH "Bias (Research)" | 8,540 |
| 5 | TI Bias OR AB Bias OR TI biases OR AB biases | 64,322 |
| 6 | S4 OR S5 | 69,382 |
| 7 | MH "Community Health Services" OR MH "Health Care Delivery" OR MH "Primary Health Care" OR MH "Healthcare Disparities" OR MH "Decision Support Systems, Clinical" OR MH "Decision Support Techniques" | 182,192 |
| 8 | TI "Decision Support" OR AB "Decision Support" OR TI ((Primary OR Community OR Health) N2 Care) OR AB ((Primary OR Community OR Health) N2 Care) OR TI ((Health OR delivery) N3 Care) OR AB ((Health OR delivery) N3 Care) OR TI Healthcare OR AB Healthcare OR TI "Health Service#" OR AB "Health Service#" OR TI (health* N2 (equit* OR disparit*)) OR AB (health* N2 (equit* OR disparit*)) | 597,750 |
| 9 | S7 OR S8 | 681,426 |
| 10 | S3 AND S6 AND S9 | 298 |
| 11 | S10 AND PY 2017-2022 | 225 |

**PsycInfo (OVID)**

**Date of the search:** 15-11-2022

**Database limit:** The results of the research strategy were limited to the last 5 years (2017-2022)

| **#** | **Search strategy** | **Results** |
| --- | --- | --- |
| 1 | Artificial Intelligence/ OR Natural Language Processing/ OR machine learning/ OR machine learning algorithms/ OR expert systems/ OR decision support systems/ OR neural networks/ OR artificial neural networks/ OR Algorithms/ | 59970 |
| 2 | Expert System?.ti,ab,id OR ((artificial OR computational) adj2 intelligence).ti,ab,id OR ((Machine? OR Supervised OR Unsupervised OR Reinforcement OR Deep) adj2 Learning).ti,ab,id OR "Neural Networks".ti,ab,id OR (AI adj (technolog* OR system? OR algorith* OR application?)).ti,ab,id OR (predicti* adj (model? OR algorithm?)).ti,ab,id OR "natural language processing".ti,ab,id | 44654 |
| 3 | 1 OR 2 | 79646 |
| 4 | Bias.hw | 16816 |
| 5 | Bias.ti,ab,id OR biases.ti,ab,id | 97239 |
| 6 | 4 OR 5 | 99469 |
| 7 | Community Health/ OR health care services/ OR primary health care/ OR Health Care Delivery/ OR Health Disparities/ OR social equity/ OR equity/ OR Decision Support Systems/ | 103345 |
| 8 | "Decision Support".ti,ab,id OR ((Primary OR Community OR Health) adj2 Care).ti,ab,id OR ((Health OR delivery) adj3 Care).ti,ab,id OR Healthcare.ti,ab,id OR "Health Service?".ti,ab,id OR (health* adj2 (equit* OR disparit*)).ti,ab,id | 258598 |
| 9 | 7 OR 8 | 287371 |
| 10 | 3 AND 6 AND 9 | 157 |
| 11 | limit 10 to yr="2017-2022" | 85 |
