# Supplementary material for: Risk of Bias Mitigation for Vulnerable and Diverse Groups in Community-Based Primary Health Care Artificial Intelligence Models: Protocol for a Rapid Review
Source: JMIR Res Protoc. 2023 Jun 26;12:e46684. doi: 10.2196/46684 (PMC10337340; doi:10.2196/46684)
Supplement: Multimedia Appendix 2 [file resprot_v12i1e46684_app2.pdf]

OBSERVATOIRE INTERNATIONAL SUR LES IMPACTS SOCIÉTAUX DE L'IA ET DU NUMÉRIQUE

Chercheurs principaux	Titre du projet	Co-chercheurs	Budget
**Marie-Pierre GAGNON (ULaval)**	Protéger et Engager les populations vulnérables dans le développement des Modèles prédictifs en soins de santé primaires pour une IA inclusive, diversifiée et équitable (PREMIA)	Jean-Sébastien PAQUETTE (ULaval)<br>Caroline RHÉAUME (ULaval)<br>Maxime SASSEVILLE (UQChicoutimi)<br>Philippe DESPRÉS (ULaval)<br>Karine GENTELET (UQO)	123 592,28 $

Evaluation 1

Critère	Appréciation	Note	Sur	Commentaire
**1. Équipe de recherche, attraction et formation de la relève scientifique et maillage entre les chercheur(e)s de l'OBVIA et leurs partenaires (35%)**	Excellent	30	/35	Il s'agit d'une équipe de 8 chercheuses et chercheurs à divers stades de carrière provenant de 3 universités québécoises et d'une université française. L'équipe de travail multidisciplinaire, multiuniversitaire et internationale est déjà en action et elle s'appuie sur une communauté vritruelle déjà constituée (COMVIP). L'importance de l'approche interdisciplinaire est frappante, notamment par le fait que tous les membres de l'équipe recevront une formation à la recherche en partenariat, basée sur la Stratégie de recherche axée sur le patient (SRAP) des IRSC et adaptée par l'Unité SOUTIEN-SRAP du Québec. Le niveau d'implication des étudiants est identifiée dans toutes les sections de la proposition, ce qui en montre l'importance. Les citoyens partenaires sont compensés, ce qui dénote la place qu'ils prennent dans l'équipe.
**2. Pertinence scientifique et sociale, précision du projet, de ses hypothèses et de sa méthodologie (35%)**	Excellent	29	/35	La pertinence scientifique et les méthodes sont bien justifiées. Je me questionne sur le bias de sélection des patients partenaires dits 'vulnérables ou marginalisés' pour un projet se déroulant en virtuel. Il semble y avoir une contradiction au niveau socio-économique ou même au niveau de l'accessibilité (ex. personnes avec déficience motrice, cognitive, langage). On ne voit pas comment ces biais seront pris en compte pour former les groupes de réflexion.
**3. Retombées du projet, rayonnement créatif et potentiel de transfert des connaissances (20%)**	Excellent	17	/20	L'échéancier est réaliste et les travaux s'appuient clairement sur l'enchainement des étapes. On remarque que le budget dédié à la mobilisation des connaissances est surtout axé sur la compensation des patients partenaires qui est un excellent moyen de mobilisation active des connaissances. Les autres activités financées dans le budget sont relativement traditionnelles (conférences scientifiques et publications libre accès). Par contre, on mentionne dans les activités du projet que les résultats des différentes phases du projet seront partagés lors d'événements grand public en ligne (ex : Dialogues centre de recherche en santé durable VITAM, exposition virtuelle) et en présentiel (café partage, rencontre d'associations civiles et de groupes communautaires). De plus, les chercheurs s'engagent à partager les résultats avec leurs partenaires de l'Unité SOUTIEN-SRAP du Québec (dont plusieurs membres de l'équipe font partie), les autres unités similaires au Canada, et leurs partenaires internationaux lors de conférences scientifiques (ex. CALASS, ISDM, SIDIIEF) et de présentations visant un plus large public (ex. réseau des bibliothèques, webinaires). Pour chaque phase du projet, un manuscrit sera soumis pour publication dans une revue scientifique à libre accès révisée par les pairs.
**4. Budget (10%)**	Excellent	9	/10	Très intéressant d'avoir prévu uen somme pour soutenir l'équipe qui mettra en place les paramètres de réduction des biais. Il aurait été souhaitable de détailler les contributions en nature et en espèces des divers partenaires dans le budget soumis pour les mettre en valeur.
		85		**Il s'agit d'un projet bien ficelé, avec un nœud de collaborateurs et une équipe bien organisée dont les objectifs sont bien définis et réalistes en fonction de l'échéancier. L'équipe a pris soin de faire ressortir le lien entre le projet et l'approche EDI. J'aurais aimé voir une définition opérationnelle des 'populations vulnérables ou marginalisées'. Est-il réaliste dans un seul projet de traiter tous les types d'exclusion? Mention à corriger au niveau du format: on retrouve quelques mentions (ref) sans la référence.**

Evaluation 2

Critère	Appréciation	Note	Sur	Commentaire
**1. Équipe de recherche, attraction et formation de la relève scientifique et maillage entre les chercheur(e)s de l'OBVIA et leurs partenaires (35%)**	Excellent	30	/35	Plusieurs universités impliquées dont une française/ profils différents: informiers, éthicie, médecin, biologiste, physicien. Le projet gagnerait à impliquer au moins un juriste spécialisé en droit du numérique (notamment parce que le sujet soulève de belles questions de discrimination, de fracture numérique et soulève des défis en termes de co-régulation Droit-secteur) ainsi qu'au moins un informaticien (notamment sur les causes et solutions des biais algorithmiques et des biais dans les données utilisées par les types de systèmes envisagés). Il est envisagé d'impliquer dans la recherche-action des personnes du terrain (citoyens représentants des catégories vulnérables). Il serait judicieux d'impliquer aussi des chercheurs qui travaillent dans des laboratoires d'IA à la recherche de solution pour diminuer les biais (par exemple les chercheurs qui recourent à des "algorithwatchers", qui sont des algorithmes capables d'identifier eux-mêmes les biais dans les données; ou des chercheurs qui pratiquent les méthodes de test en laboratoire, une équipe confectionne l'algorithme, l'autre équipe l'évalue).
**2. Pertinence scientifique et sociale, précision du projet, de ses hypothèses et de sa méthodologie (35%)**	Excellent	32	/35	Sujet éminemment important d'un point de vue sociétal et responsable. Sujet bien anglé, qui donne confiance pour l'aboutissement de celui-ci car la recherche a un objet clair, ciblé, qui le rend réalisable dans le délai imparti. Sujet peu étudié en matière d'IA qui se concentre peu sur les vulnérabilités qui en découlent.
**3. Retombées du projet, rayonnement créatif et potentiel de transfert des connaissances (20%)**	Excellent	17	/20	Rôle sociétal fort puisqu'il s'agira de formuler des recommandations visant les personnes vulnérables, catégorie sociale souvent délaissée lorsqu'il est question d'IA. / pour la visibilité: événements publics, attraction de chercheurs, mise en réseau Canada-France/partie du budget allouée à la réalisation d'un doctorat (impact sur les étudiants et développement de l'expertise à moyen terme)
**4. Budget (10%)**	Excellent	10	/10	Les postes budgétaires sont détaillés et semblent pertinents et adéquats par rapport aux différentes missions du projet.
		89		

Evaluation 3

Critère	Appréciation	Note	Sur	Commentaire
**1. Équipe de recherche, attraction et formation de la relève scientifique et maillage entre les chercheur(e)s de l'OBVIA et leurs partenaires (35%)**	Excellent	35	/35	Constituée de 8 personnes hautement qualifiées dans des savoirs variés, l'équipe de recherche pluridisciplinaire et également internationale me semble bien construite et parfaitement à même de mener à bien ce projet. Ce dernier s'inscrit bien dans les axes stratégiques de l'OBVIA.
**2. Pertinence scientifique et sociale, précision du projet, de ses hypothèses et de sa méthodologie (35%)**	Excellent	33	/35	Ce projet de recherche, appliqué aux questions de prédictibilité en santé, veut œuvrer à la réduction des biais algorithmiques et à l'éducation à l'IA pour la population distante et vulnérable. Sans être vraiment original, le sujet me semble important car il concerne un secteur d'activité où l'IA est déjà en train de transformer les pratiques professionnelles et les services publics de santé.
**3. Retombées du projet, rayonnement créatif et potentiel de transfert des connaissances (20%)**	Qualité démontrée	15	/20	L'objectif du projet étant de "mieux protéger les populations vulnérables en contexte de soins de santé de première ligne et santécommunautaire", les résultats attendus sont des indicateurs, des mesures et des recommandations concernant les biais potentiels de l'IA prédictive.
**4. Budget (10%)**	Excellent	9	/10	Le buget, d'un montant global de 123 592 $ sur 3 ans, me semble bien correspondre à l'ambition scientifique du projet, il apparait raisonnable et bien construit.
		92		**Du fait de ses qualités énumérées plus haut, ce projet me semble mériter le financement.**
